# Role of Human Papilloma Virus and Lifestyle Factors in Overall Survival of Patients with Oropharyngeal Squamous Cell Carcinoma

**DOI:** 10.3390/medicina58040557

**Published:** 2022-04-18

**Authors:** Daisuke Nishikawa, Nobuhiro Hanai, Taijiro Ozawa, Tadashi Kitahara, Yasuhisa Hasegawa

**Affiliations:** 1 Department of Otolaryngology-Head and Neck Surgery, Nara Medical University, 840 Shijocho, Kashihara 634-8522, Nara, Japan; daidaidadai@gmail.com (D.N.); tkitahara@naramed-u.ac.jp (T.K.); 2 Department of Head and Neck Surgery, Aichi Cancer Center Hospital, 1-1 Kanokoden, Chikusaku, Nagoya 464-8681, Aichi, Japan; 3 Department of Otolaryngology, Toyohashi Municipal Hospital, 50 Hakken-Nishi, Aotakecho, Toyohashi 441-8570, Aichi, Japan; ozawa-taijiro@toyohashi-mh.jp; 4 Department of Head and Neck Surgery, Asahi University Hospital, 3-23 Hashimotocho, Gifu 500-8523, Gifu, Japan; yhasegawa@dent.asahi-u.ac.jp

**Keywords:** oropharyngeal squamous cell carcinoma, HPV, smoking history, alcohol consumption

## Abstract

*Background:* Patients with human papillomavirus (HPV)-associated oropharyngeal squamous cell carcinoma (OPSCC) have a significantly better treatment response and overall survival (OS) rates than non-HPV-associated OPSCC. *Objectives***:** We conducted the present study to further characterize the interplay between lifestyle risk factors, which are not only HPV status, but also smoking history and alcohol consumption, and the OS to optimize the treatment of patients with OPSCC. *Materials and Methods:* Between January 2006 and December 2013, 94 patients newly diagnosed with OPSCC were treated with curative intent at Aichi Cancer Center Hospital (Nagoya, Japan). To determine negative prognostic factors associated with the OS, univariate and multivariable Cox regression analyses were performed. *Results:* Of the 94 OPSCC patients, 53 (56.4%) were positive for HPV. The univariate analysis revealed that T classification, smoking history, alcohol consumption, and HPV status were significant determinants of the OS. In the multivariate analysis, adjusted for the clinical stage, smoking history, alcohol consumption, HPV status, and a smoking history of >10 pack-years was an independent negative prognostic factor for the OS among patients with OPSCC (HR: 10.4, 95 %CI: 1.34–80.6, *p* < 0.05). *Conclusions:* Smoking is a very important negative prognostic factor even in cases of HPV-associated OPSCC. The impact of smoking needs to be reaffirmed when deciding on treatment plans and de-escalation trials in OPSCC, even in cases of HPV-associated OPSCC.

## 1. Introduction

Head and neck squamous cell carcinoma (HNSCC) is a heterogeneous disease that includes cancers involving the oral cavity, pharynx, and larynx. Worldwide, there are more than 550,000 new cases of HNSCC each year and 380,000 associated deaths [1]. Tobacco smoking and excessive alcohol consumption have traditionally been considered the main risk factors for HNSCC [2,3].

Human papillomavirus (HPV)-associated oropharyngeal squamous cell carcinoma (OPSCC) has been established as a distinct clinical entity with favorable patient outcomes compared to other types of HNSCC, which are commonly associated with heavy tobacco and alcohol use. Patients with HPV-associated head and neck cancer have a significantly better treatment response and overall survival (OS) rates, irrespective of age, sex, or T classification, than non-HPV-associated OPSCC [4,5]. However, this significant survival advantage is not homogeneous, and several studies have suggested that among HPV-positive patients, those with a smoking history had worse oncological outcomes and a significantly increased risk of death [4,6,7,8,9,10,11]. 

In Japan, studies showed the prevalence rate of HPV in OPSCC cases to be 40–50% over the last 2 decades [12,13], suggesting an increasing trend of HPV-associated OPSCC cases in this region. In a multi-institutional observational study based on the Head and Neck Cancer Registry of Japan (2011–2014), among HPV-positive patients, those with a ≤ 10 pack-years smoking history comprised approximately 37.7% and nondrinkers comprised approximately 24.7% [14].

Because of the promising prognosis of HPV-positive OPSCC, head and neck oncologists are actively exploring ways to limit treatment-related toxicities by reducing the number of treatment modalities and/or reducing the intensity/dose of a given modality without compromising efficacy. The proper identification of prognostic groups is essential for allowing safe de-escalation strategies to be implemented [15]. We therefore conducted the present study to further characterize the interplay between lifestyle risk factors, which were not only HPV status but also smoking history and alcohol consumption, and the OS to optimize the treatment of patients with OPSCC.

## 2. Materials and Methods

### 2.1. Patients

Between January 2006 and December 2013, 99 patients newly diagnosed with OPSCC were treated with curative intent at the Department of Head and Neck Surgery, Aichi Cancer Center Hospital. Among these patients, five were excluded due to a lack of data for their history of tobacco smoking and alcohol consumption. A total of 94 patients were thus enrolled in the study, which was approved by the review board of our institution. All of the patients gave their informed consent for the treatment and examination. 

The anatomical locations from which the tissue samples were obtained are shown in Table 1.

### 2.2. HPV and Cancer Diagnoses

HPV status was confirmed in all patients via genotyping of HPV. The samples were examined by pathologists at Aichi Cancer Center Hospital and assigned a histologically confirmed diagnosis of squamous cell carcinoma.

### 2.3. Detection and Genotyping of HPV

Genomic DNA was isolated from biopsy samples and collected in a liquid-based cytology medium. HPV-DNA testing was performed using the multiplex PCR method (PapiPlex) at the GLab Pathology Center Co., Ltd. (Sapporo, Japan). This can detect 16 high- and low-risk HPV genotypes (genotypes 6, 11, 16, 18, 30, 31, 33, 35, 39, 45, 51, 52, 56, 58, 59, and 66) in a single tube. Nishiwaki et al. provided more details about the multiplex PCR method (PapiPlex) in their paper [15]. In brief, HPV-genotype-specific primers were designed on the basis of multiplex-sequence alignments. PCR was performed with a multiplex PCR kit (Qiagen Inc., Valencia, CA, USA), according to the manufacturer’s instructions, with minor modifications. The HPV genotypes in the samples were identified based on the sizes of the amplicons.

### 2.4. Staging and Treatment

The clinical TNM classification was determined with a routine physical examination, flexible pharyngeal endoscopy, enhanced computed tomography or magnetic resonance imaging, and ^18^F-fluorodeoxyglucose-positron emission tomography/computed tomography, if possible. The TNM staging system was based on the Union for International Cancer Control Classification of Malignant Tumors (Seventh edition). The patient distribution according to the TNM classification is shown in Table 2.

All cases were managed by a multidisciplinary tumor board to determine whether the primary treatment course should be surgery or definitive radiotherapy/chemoradiotherapy. HPV status was not considered in treatment decisions because this study was conducted without disclosing the HPV status to the clinician and it was conducted between January 2006 and December 2013, before the publication of the eighth edition of the TNM staging system.

These 94 patients were classified into the surgery group or radiotherapy (RT) group according to the primary tumor treatment modality. The surgery group was treated with curative surgery ± RT with or without chemo/biotherapy (*n* = 24). The RT group was treated with definitive RT at a total dose 60–70 Gy, with 2 Gy per fraction with or without chemo/biotherapy (*n* = 70). Among the patients in the RT group, 55 patients were treated with concomitant platinum-based chemoradiotherapy, and 2 patients were treated with concomitant cetuximab-based bioradiotherapy, while 13 patients were treated with RT alone. In regimens of concurrent chemoradiotherapy, cisplatin was delivered on a weekly (25–30 mg/m^2^/day for three to seven cycles) (*n* = 39), every three-week (80–100 mg/m^2^/day for two to three cycles) (*n* = 14), or a weekly (50 mg/m^2^/day for four cycles by intra-arterial) (*n* = 1) schedule, or carboplatin was delivered on a weekly (for three cycles) (*n* = 1) schedule. The regimen of concurrent bioradiotherapy was cetuximab at 400 mg/m^2^ on day 1 of the week preceding RT and a weekly dose of 250 mg/m^2^ cetuximab during RT.

Cisplatin-based induction chemotherapy, whjich comprises a combination of cisplatin and 5-fluorouracil, was indicated for 67 of the 94 patients. Fifty-five good responders were treated with RT with or without chemo/biotherapy instead of surgery and were classified into the RT group. Another 12 patients were treated with surgery and were classified into the surgery group. 

### 2.5. Consumption of Alcohol and Tobacco

All patients were interviewed about their daily amount and duration of tobacco smoking and alcohol consumption during the first medical examination at our department. The smoking history was then calculated in total pack-years (20 cigarette-years).

### 2.6. Statistical Analyses

The primary outcome was the OS, defined as the time from the date of any therapy initiation to death from any cause. Actuarial rates of the OS were estimated using the Kaplan–Meier method. The log-rank test was used to compare the OS between the two groups. A Cox proportional hazards model was used to estimate the hazard ratios (HRs) and 95% confidence intervals (CIs). *p* values of <0.05 were considered to indicate statistical significance.

## 3. Results

### 3.1. HPV Status and Patient Characteristics

Of the 94 OPSCC patients, 53 (56.4%) were positive for HPV. Table 3 summarizes the relevant clinical data of the 94 OPSCC patients. Among HPV-positive patients, the cumulative pack-years of tobacco smoking were significantly lower than those among HPV-negative patients (*p* < 0.01). The alcohol consumption among HPV-positive patients was also significantly lower than that among HPV-negative patients (*p* = 0.028).

### 3.2. Survival Outcomes

**Figure 1:**
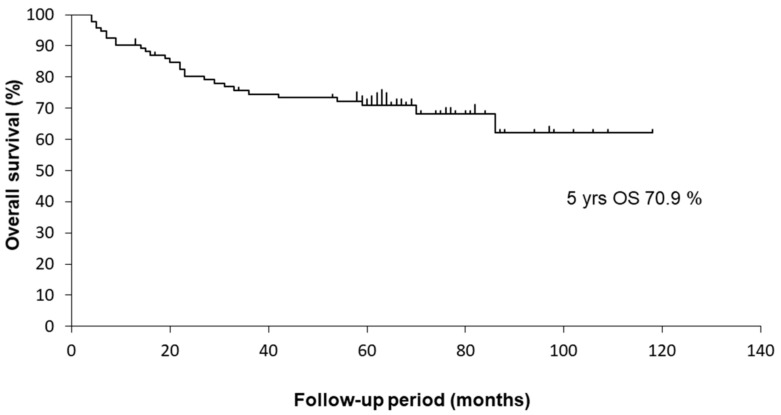
Kaplan–Meier curves for OS of 94 OPSCC patients.

The median follow-up period of the surviving patients was 55.3 (range 4.5–120.2) months. There were 28 deaths, including 18 from primary cancer and 10 from other causes. The 5-year OS rate of the 94 OPSCC patients was 70.9%. The Kaplan–Meier curves are shown in Figure 1. 

**Figure 2:**
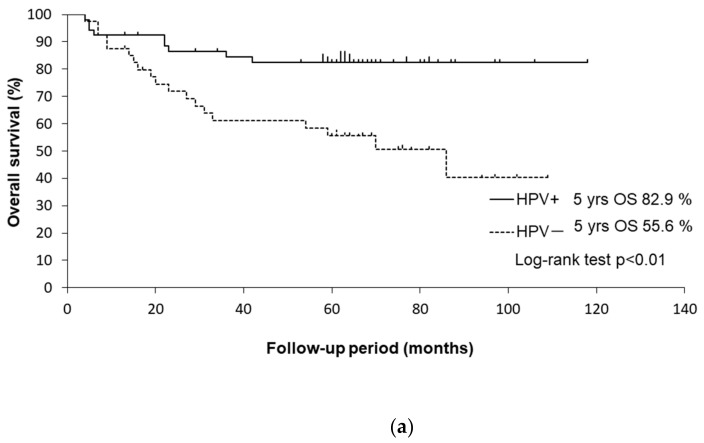

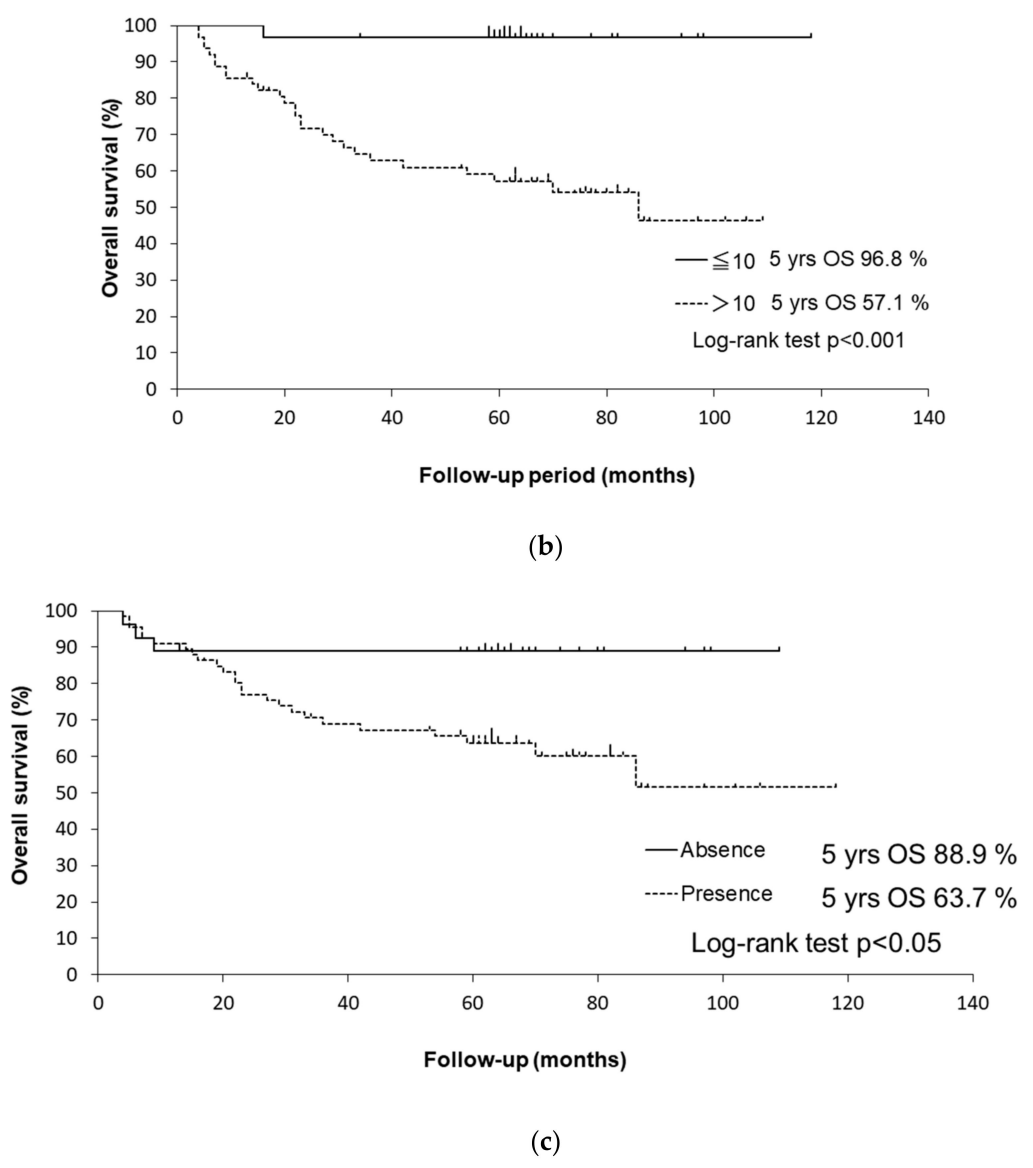
(**a**) Kaplan–Meier curves for OS of HPV-positive and HPV-negative patients. (**b**) Kaplan–Meier curves for OS of patients with a ≤ 10 and with a > 10 pack-years smoking history. (**c**) Kaplan–Meier curves for OS of patients with/without alcohol consumption.

The univariate analysis of factors associated with the OS is shown in Table 4. The univariate analysis revealed that HPV status, smoking history, alcohol consumption, and the T classification were significant determinants of the OS. The Kaplan–Meier curves are shown in Figure 2.

The 5-year OS rates were 82.4% in HPV-positive patients and 55.6% in HPV-negative patients (*p* < 0.01 by log-rank test). HPV-negative patients had a significantly worse OS than HPV-positive patients (HR: 3.18, 95% CI: 1.43–7.04, *p* < 0.001). 

**Figure 3:**
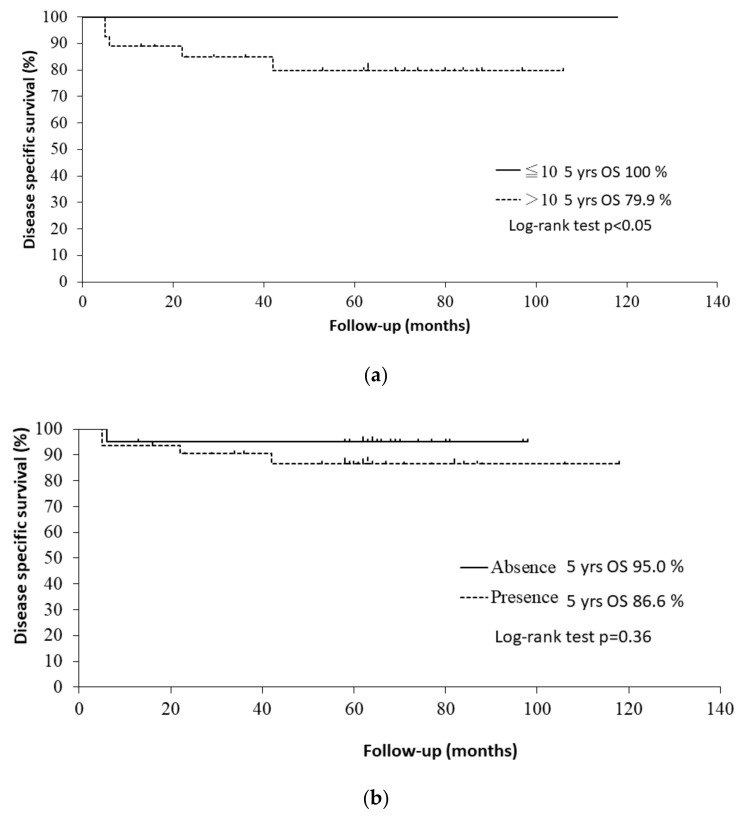
(**a**) Kaplan–Meier curves for disease-specific survival of HPV-positive patients with a ≤ 10 and with a > 10 pack-years smoking history. (**b**) Kaplan–Meier curves for disease-specific survival of HPV-positive patients with/without alcohol consumption.

The 5-year OS rates were 96.8% in patients with a ≤ 10 pack-years smoking history and 57.1% in patients with a > 10 pack-years smoking history (*p* < 0.001 by log-rank test). Patients with a > 10 pack-years smoking history had a significantly worse OS than those with a ≤ 10 pack-years smoking history (HR: 17.5, 95% CI: 2.37–129, *p* < 0.01). Regarding the stratification by smoking history in HPV-positive patients, the 5-year disease-specific survival rates were 100% in patients with a ≤ 10 pack-years smoking history and 79.9% in those with a > 10 pack-years smoking history (*p* < 0.05 by log-rank test). The Kaplan–Meier curves are shown in Figure 3a. Former and current smokers had a significantly worse OS than never smokers (HR: 12.4, 95% CI: 1.69–12.4, *p* < 0.05). Former smokers (HR: 10.3, 95% CI: 1.32–80.1, *p* < 0.05) and current smokers (HR: 13.7, 95% CI: 1.82–103.7, *p* < 0.05) had a significantly worse OS than never smokers.

The 5-year OS rates were 88.9% in patients with alcohol consumption and 63.7% in patients who did not consume alcohol (*p* < 0.05 by log-rank test). Patients with alcohol consumption had a significantly worse OS than those who did not consume alcohol in the univariate analysis (HR: 3.67, 95% CI: 1.11–12.2, *p* < 0.05). Regarding the stratification by alcohol consumption in HPV-positive patients, the 5-year disease-specific survival rates were 95.0% in patients with alcohol consumption and 86.6% in those who did not consume alcohol (*p* = 0.36 by log-rank test). The Kaplan–Meier curves are shown in Figure 3b.

There was no significant difference in the OS between the surgery group and the RT group in the univariate analysis (HR: 0.85, 95% CI: 0.38–1.89, *p* = 0.69). Induction chemotherapy was not a significant determinant of the OS in patients with OPSCC in the univariate analysis (HR: 1.27, 95% CI: 0.54–3.00, *p* = 0.58). 

In the multivariate analysis, adjusted for the clinical stage, smoking history, alcohol consumption, and HPV status, a smoking history of >10 pack-years was an independent negative prognostic factor for the OS in patients with OPSCC (HR: 10.4, 95% CI: 1.34–80.6, *p* < 0.05). The results of the multivariate analysis of factors associated with the OS are shown in Table 5.

## 4. Discussion

In this study, the T classification, smoking history, alcohol consumption, and HPV status were shown to be significant determinant of the OS in patients with OPSCC in the univariate analysis. Among the lifestyle risk factors, those associated with a shorter OS in patients with OPSCC were as follows (in order of impact): a smoking history > 10 pack-years, HPV status, and alcohol consumption [16]. 

HPV status is recognized as the strongest and most significant prognostic factor in patients with OPSCC. HPV-associated OPSCC has been already classified as the disease that is different from non-HPV-associated OPSCC. In this study, the HPV status was a strong prognostic factor in the univariate analysis (HR: 3.18, 95% CI: 1.43–7.04, *p* < 0.001) and in the multivariate analysis (HR: 2.1, 95% CI: 0.91–4.65, *p* = 0.08). Because the study population was relatively small, HPV status was marginal for a level of significance in the multivariate analysis (*p* = 0.08).

Regarding the relationship with the OS according to the multivariate analysis of these lifestyle risk factors, it was reconfirmed that a smoking history >10 pack-years was the strongest and most significant prognostic factor (HR: 10.4, 95% CI: 1.34–80.6, *p* = 0.03). These results might be because the detrimental effects of smoking on health compromise treatment tolerance and increase the risk of death from other conditions, particularly cardio-vascular and pulmonary diseases.

The HPV status and tobacco exposure (≤10 or >10 pack-years) were the strongest determinants of the survival in patients with OPSCC [4]. A smoking habit at or before the cancer diagnosis was a significant determinant of the OS. For patients with early-stage HNSCC, the risk of death has been associated with the smoking status at the diagnosis and exposure to tobacco as measured in smoking duration (years) and intensity and duration (pack-years). A dose-dependent (according to the smoking intensity (cigarettes/day)) increase in the risk of dying was also observed, although the test for linear trend was not statistically significant [17]. 

Our results indicated an association between the smoking status/smoking history and the OS in patients with OPSCC. The presence of a smoking history, the smoking status at the diagnosis, and a smoking history of >10 pack-years were significant determinants of the OS. The following factors were associated with a shorter OS in patients with OPSCC (in order of impact): a smoking history >10 pack-years, current smoking at the diagnosis, and the presence of a smoking history. Regarding the smoking status/smoking history, a smoking history >10 pack-years was the strongest prognostic factor for the OS.

This study also showed that the treatment modality for primary tumor and induction chemotherapy were not significant determinants of survival in patients with OPSCC. Some studies have similarly demonstrated comparable OS rates with primary surgery and primary RT [18,19,20]. 

Patients with OPSCC receive multimodality treatments, including radiotherapy/chemoradiotherapy or surgery followed by radiotherapy with or without chemo/biotherapy. As the number of treatment options grows, patients and physicians face increasingly complex decisions, complicated by uncertainty concerning treatment effectiveness as well as the potential benefits and harms [21]. HPV-associated head and neck cancer patients have a significantly better treatment response and better OS rates than non-HPV-associated head and neck cancer patients [4,5]. For these reasons, there are many ongoing clinical trials attempting to de-escalate treatment in order to minimize long-term morbidity, including dysphagia, xerostomia, chronic aspiration, and chronic fatigue, without compromising disease control. 

Several limitations associated with the present study warrant mention. First, the study population was relatively small. For this reason, it was not possible to analyze each stage. Second, we lacked information about the expression of p16, as the p16 expression was not routinely examined prior to treatment in our institution at the time. Third, we did not use HPV RNA to demonstrate active infection for HPV status determination. Only PCR results showing the presence of HPV DNA do not conclusively indicate HPV-associated OPSCC, as HPV sometimes exists in the head and neck region as a bystander entity.

The results from this study should be interpreted with these limitations in mind. Nevertheless, we believe that smoking history and HPV status are strong prognostic factors in OPSCC and may be useful as markers for optimizing the treatment of patients with OPSCC.

## 5. Conclusions

Even in the case of HPV-related OPSCC, lifestyle factors such as smoking history are significantly negative prognostic factors. This study reaffirmed the importance of stratifying the HPV-positive population by tobacco use when deciding on treatment plans or de-escalation trials for OPSCC. We believe that this study provides useful evidence for future clinical trials.

## Figures and Tables

**Table 1:** Subsite.

	Total *n* = 94	HPV Status	
		Positive *n* = 53	Negative *n* = 41
	No. (%)	No. (%)	No. (%)
Lateral wall	64 (68)	40 (75)	24 (59)
Anterior wall	23 (25)	13 (25)	10 (24)
Posterior wall	4 (4)	0 (0)	4 (10)
Superior wall	3 (3)	0 (0)	3 (7)

**Table 2:** Distribution of 94 patients according to TNM classification.

Tclassification	N0	N1	N2	N3	Total
T1	1	0	2	0	3 (3%)
T2	8	9	33	1	51 (54%)
T3	1	4	10	3	18 (19%)
T4	2	3	15	2	22 (24%)
Total	12 (13%)	16 (17%)	60 (64%)	6 (6%)	94

**Table 3:** Clinical parameters.

	Total *n* = 94	HPV Status			No. of Pack-Years			Alcohol Consumption		
		Positive *n* = 53	Negative *n* = 41		≤10 *n* = 31	>10 *n* = 63		Presence *n* = 67	Absence *n* = 27	
	No. (%)	No. (%)	No. (%)	*p* Value	No. (%)	No. (%)	*p* Value	No. (%)	No. (%)	*p* Value
Age, range (median) years	35–79 (59)	35–79 (58)	37–78 (60)	0.20 ^b^	37–76 (57)	35–79 (60)	0.37 ^b^	35–78 (59)	41–79 (59)	0.95 ^b^
Sex				0.71 ^a^			<0.01 ^a^			0.04 ^a^
Male	75 (80)	43 (81)	32 (78)		17 (55)	58 (92)		57 (85)	18 (67)	
Female	19 (20)	10 (19)	9 (22)		14 (45)	5 (8)		10 (15)	9 (33)	
Clinical T classification				0.28 ^a^			0.01 ^a^			0.81 ^a^
T1–2	54 (57)	33 (62)	21 (51)		24 (77)	30 (48)		39 (58)	15 (56)	
T3–4	40 (43)	20 (38)	20 (49)		7 (23)	33 (52)		28 (42)	12 (44)	
Clinical N classification				0.27 ^a^			0.01 ^a^			0.08 ^a^
N0	12 (13)	5 (9)	7 (17)		8 (26)	4 (6)		6 (9)	6 (22)	
N1–3	82 (87)	48 (91)	34 (83)		23 (74)	59 (94)		61 (91)	21 (78)	
Clinical stage				0.14 ^a^			<0.01 ^a^			0.06 ^a^
Stage I–II	9 (10)	3 (6)	6 (15)		6 (19)	3 (5)		4 (6)	5 (19)	
Stage III–IV	85 (90)	50 (94)	35 (85)		25 (81)	60 (95)		63 (94)	22 (81)	
Tobacco smoking, range (median) pack-years	0–88(29)	0–83(21)	0–88(38)	<0.01 ^b^	0–10 (1)	12–88 (42)		0–88(35)	0–60 (14)	<0.01 ^b^
No. of pack-years				<0.01 ^a^						<0.01 ^a^
≤10	31 (33)	25 (47)	6 (15)					15 (22)	16 (59)	
>10	63 (67)	28 (53)	35 (85)					52 (78)	11 (41)	
Alcohol consumption				0.03 ^a^			<0.01 ^a^			
Presence	67 (71)	33 (62)	34 (83)		15 (48)	52 (83)				
Absence	27 (29)	20 (38)	7 (17)		16 (52)	11 (17)				
HPV status							<0.01 ^a^			0.03 ^a^
Positive	53 (56)				25 (81)	28 (44)		33 (49)	20 (74)	
Negative	41 (44)				6 (19)	35 (56)		34 (51)	7 (26)	
Treatment group				0.49 ^a^			0.36 ^a^			0.07 ^a^
Surgery	24 (26)	11 (21)	13 (32)		9 (29)	15 (24)		21 (31)	3 (11)	
Radiation	70 (74)	42 (79)	28 (68)		22 (71)	48 (76)		46 (69)	24 (89)	
Induction chemotherapy				0.57 ^a^			0.13 ^a^			0.53 ^a^
Presence	67 (71)	39 (74)	28 (68)		19 (61)	48 (76)		49 (72)	18 (67)	
Absence	27 (29)	14 (26)	13 (32)		12 (39)	15 (24)		18 (27)	9 (33)	

^a^ Chi-square test. ^b^ Mann–Whitney U test.

**Table 4:** The univariate analysis of factors associated with OS.

		HR	95% CI	*p*
Age	>50 vs. ≤50	1.61	0.55–4.65	0.38
Sex	Male vs. Famale	4.02	0.95–16.9	0.06
Clinical T	T3-4 vs. T1-2	2.87	1.32–6.25	0.01
Clinical N	N1-3 vs. N0	2.23	0.53–9.41	0.27
Clinical stage	III-IV vs. I-II	3.62	0.49–26.6	0.21
Smoking (no. of pack-years)	>10 vs. ≤10	17.5	2.37–129.0	0.01
Alcohol consumption	Presence vs. Absence	3.67	1.11–12.2	0.03
HPV status	Negative vs. Positive	3.18	1.43–7.04	<0.01
Treatment group	Surgery vs. Radiation	0.85	0.38–1.89	0.69
Induction chemotherapy	Presence vs. Absence	1.27	0.54–3.00	0.58

Cox proportional hazards models were used to estimate hazard ratios (HRs) and 95% confidence intervals (CIs).

**Table 5:** The multivariate analysis of factors associated with OS.

		HR	95% CI	*p*
Clinical stage	III–IV vs. I–II	2.6	0.34–19.4	0.36
Smoking (no. of pack-years)	>10 vs. ≤10	10.4	1.34–80.6	0.03
Alcohol consumption	Presence vs. Absence	1.6	0.48–5.50	0.44
HPV status	Negative vs. Positive	2.1	0.91–4.65	0.08

Cox proportional hazards models were used to estimate hazard ratios (HRs) and 95% confidence intervals (CIs).
